# Uptake, safety and effectiveness of inactivated influenza vaccine in inflammatory bowel disease: a UK-wide study

**DOI:** 10.1136/bmjgast-2024-001370

**Published:** 2024-06-18

**Authors:** Georgina Nakafero, Matthew J Grainge, Tim Card, Christian D Mallen, Jonathan S Nguyen Van-Tam, Abhishek Abhishek

**Affiliations:** 1 Academic Rheumatology, University of Nottingham, Nottingham, UK; 2 Nottingham NIHR BRC, Nottingham, UK; 3 Lifespan and Population Health, University of Nottingham, Nottingham, UK; 4 Primary Care Centre Versus Arthritis, Keele University, Keele, UK

**Keywords:** INFLAMMATORY BOWEL DISEASE, CROHN'S COLITIS, ULCERATIVE COLITIS, EPIDEMIOLOGY

## Abstract

**Objective:**

To investigate (1) the UK-wide inactivated influenza vaccine (IIV) uptake in adults with inflammatory bowel disease (IBD), (2) the association between vaccination against influenza and IBD flare and (3) the effectiveness of IIV in preventing morbidity and mortality.

**Design:**

Data for adults with IBD diagnosed before the 1 September 2018 were extracted from the Clinical Practice Research Datalink Gold. We calculated the proportion of people vaccinated against seasonal influenza in the 2018–2019 influenza cycle. To investigate vaccine effectiveness, we calculated the propensity score (PS) for vaccination and conducted Cox proportional hazard regression with inverse-probability treatment weighting on PS. We employed self-controlled case series analysis to investigate the association between vaccination and IBD flare.

**Results:**

Data for 13 631 people with IBD (50.4% male, mean age 52.9 years) were included. Fifty percent were vaccinated during the influenza cycle, while 32.1% were vaccinated on time, that is, before the seasonal influenza virus circulated in the community. IIV was associated with reduced all-cause mortality (aHR (95% CI): 0.73 (0.55,0.97) but not hospitalisation for pneumonia (aHR (95% CI) 0.52 (0.20–1.37), including in the influenza active period (aHR (95% CI) 0.48 (0.18–1.27)). Administration of the IIV was not associated with IBD flare.

**Conclusion:**

The uptake of influenza vaccine was low in people with IBD, and the majority were not vaccinated before influenza virus circulated in the community. Vaccination with the IIV was not associated with IBD flare. These findings add to the evidence to promote vaccination against influenza in people with IBD.

**WHAT IS ALREADY KNOWN ON THIS TOPIC**

- Inactivated influenza vaccine is recommended to people with inflammatory bowel disease (IBD) treated with immune-suppressing drugs.
- Concerns about influenza vaccine causing IBD flare and the lack of data on the effectiveness of influenza vaccine in people with IBD are barriers to seasonal influenza vaccination in this population.

**WHAT THIS STUDY ADDS**

- Seasonal influenza vaccination was not associated with IBD flare.
- Seasonal influenza vaccination uptake was low in people with IBD.

**HOW MIGHT THIS STUDY AFFECT RESEARCH, PRACTICE, OR POLICY**

- These findings add to the evidence to promote vaccination in people with IBD.
- Further research is needed to ascertain the vaccine uptake in the post COVID-19 pandemic era, to evaluate its clinical effectiveness in larger studies and to better understand its safety in people with IBD treated with biologics.

## Introduction

Inflammatory bowel disease (IBD) is a common immune-mediated inflammatory disease which affects 1.4% of adults in the UK.^1^ People with IBD are at an increased risk of influenza and its complications such as hospitalisation and death.^2^ Consequently, annual vaccination with the inactivated influenza vaccine (IIV) is recommended in this population if immunosuppressed due to treatment.^3 4^ Despite the recommendation for vaccination, data on the uptake, safety and effectiveness of the IIV in IBD are sparse.

In the USA, the uptake of the IIV was 48.4% in a cross-sectional survey of 951 people with IBD.^5^ A cross-sectional survey of 88 IBD people prescribed immune-suppressing drugs from a UK gastroenterology outpatient clinic found that 61.4% were vaccinated against influenza,^6^ but in another survey of 89 people with IBD, only 28.1% were vaccinated against influenza during the H1N1 pandemic in 2009.^7^ In North America and Europe, influenza vaccine uptake was low, between 28.7% and 34%.^8 9^

In terms of the association between vaccination against influenza and IBD flare, a systematic review reported that 3% of people with IBD experienced an IBD flare after vaccination against influenza; however, none of the included studies had a control group and it is unclear whether vaccination against influenza was temporally associated with an IBD flare, whether this was coincidental or an ascertainment bias.^10^

Similarly, while the IIV was as immunogenic in the IBD population as in healthy adults,^11^ the effectiveness of IIV in people with IBD has not been evaluated. In immunosuppressed adults with rheumatic disease, IIV protected against influenza-like illness (ILI), hospitalisation due to pneumonia and death due to pneumonia with a vaccine effectiveness (VE) of 30% to 50%.^12^ Vaccination was associated with a 9% lower rate of primary-care consultation for joint pain at 90 days post-vaccination.^13^

A lack of knowledge about VE and concerns about safety underlie vaccine hesitancy in people with inflammatory conditions.^14^ To provide evidence to improve the uptake of vaccination against influenza in IBD, this study aimed to assess the uptake, safety and effectiveness of the IIV in preventing ILI, lower respiratory tract infections (LRTI), pneumonia and death in people with IBD.

## Methods

### Data source

Data from the Clinical Practice Research Datalink (CPRD) Gold were used in this study. Incepted in the year 1987, CPRD Gold is an anonymised longitudinal database of electronic health records of >14 million people in the UK. CPRD participants are representative of the UK population in age, sex and ethnicity.^15^ CPRD includes information on demographics, lifestyle factors, diagnoses stored as Read codes—a coded thesaurus of clinical terms, primary-care prescriptions and immunisations. Vaccination and date of vaccination are also recorded. The data are enhanced by linkage with hospitalisation (Hospital Episode Statistics (HES)) and mortality records (Office of National Statistics) in England.

### Approval

CPRD Research Data governance (Reference 21_000614).

### Study design and period

Cross-sectional, cohort and self-controlled case series (SCCS) study designs were used to examine the IIV uptake, safety and effectiveness respectively.

The study period was from 01/09/2018 to 31/08/2019, that is, at the start and end of the 2018–2019 influenza cycle.

### Population

Adults aged ≥18 years with one or more primary care consultations for IBD and at least one prescription of steroid-sparing drugs within 12 months before the study started, i.e. 1 September 2018 were included. We selected a broad range of drugs that may be used to treat IBD, that is, 5-aminosalicylates (5-ASA) (mesalazine, balsalazide, or olsalazine), azathioprine, mercaptopurine, methotrexate, mycophenolate, ciclosporin, tacrolimus, and sirolimus for case definition. Those prescribed 5-ASAs were included as they are likely to be treated with recurrent courses of corticosteroids, making them eligible for vaccination.^16^

### Exposure

Vaccination with the IIV was defined using product and Read codes.^17^ Dates of vaccination were extracted from the CPRD.

### Outcomes

#### *Uptake:* IIV administration

*Effectiveness*: (1) Primary care consultation for lower respiratory infections (LRTI) was defined as primary-care consultation for this illness ascertained using Read codes and antibiotic prescription occurring on the same date, (2) primary care consultation for ILI ascertained using Read codes, (3) hospitalisation for pneumonia ascertained using ICD codes in the linked HES dataset as previously described 12 and (4) all-cause death. After feasibility assessment, death due to pneumonia was not selected as an outcome due to 11 events occurring in the study period.

*Safety*: IBD flare was the outcome of interest. It was defined as a new primary-care prescription of corticosteroids or 5-aminosalicylate prescription after a 4-month gap, a validated approach for ascertaining flare in IBD.^18^ To further improve the positive predictive value, we excluded participants with a record of an alternative indication for corticosteroids on the same date as consultation for IBD flare.

### Covariates

*Uptake: A*ge, gender, immune suppressive drug (yes/no) and the presence of additional indication for vaccination as specified in the Green book (yes/no).^16^ Briefly, these included chronic heart diseases, chronic respiratory diseases, chronic kidney diseases, chronic liver diseases, chronic neurological diseases, immunosuppression, diabetes and asplenia.

*Effectiveness:* A propensity score (PS) for vaccination was calculated and included as a covariate because participants at risk of influenza are more likely vaccinated.^19^ The PS included factors that account for confounding by indication, at-risk conditions, Charlson comorbidity index and health-seeking behaviour as published previously.^12^

*Safety* As weather might exacerbate IBD,^20^ the season was a covariate of interest, defined in line with the Meteorological Office: spring (1 March to 31 May), summer (1 June to 31 August), autumn (1 September to 30 November) and winter (1 December to 28 February).

### Follow-up

Participants were followed up from either 1 September 2018 or the date of registration in the current GP-surgery, whichever was the latest to the earliest date of death, date of the last data collection, transfer out of the GP-surgery or 31 August 2019 in the vaccine uptake and safety analyses. In VE analyses, follow-up was also censored at the outcome date if it occurred earlier than the study end date.

### Statistical analyses

*IIV uptake* The percentage and 95% CI of participants that received an IIV between the start and end of the 2018–2019 influenza cycle, and in-time before influenza circulated in the community (3 November 2018 as per the weekly Public Health England (PHE), now UK Health Security Agency (UKHSA) bulletins was calculated. The proportion of vaccinated individuals was stratified by age (<45, 45–64, and ≥65 years), the presence of additional indications for vaccination^16^ and immune-suppressive drug prescription in 3 months immediately before the start of the influenza cycle. Poisson regression was used to examine the multivariable association between age, sex, immune-suppressing drug use, presence of at-risk condition and receiving the IIV.

*IIV effectiveness* Mean (SD), n (%) and standardised difference (*d*) were used to examine the covariate balance between vaccinated and unvaccinated participants. PS for vaccination was calculated using logistic regression, treating vaccination status as the dependent variable. Multiple imputation using chained equation was used to impute missing data on smoking, alcohol and body mass index (BMI). Ten imputations were carried out.

Cox regression was used to calculate hazard ratios (HRs) and 95% CIs, combined using Rubin’s rule across the imputed datasets, with vaccination as the exposure of interest. Vaccination was treated as a time-varying exposure whereby the period from date of vaccination was considered as exposed, while the period before this contributed to the unexposed period. Participants without a vaccination record in the influenza cycle were considered unexposed for the entire duration. Inverse-probability treatment weighting (IPTW) using the PS was performed to account for confounding.

As IIV most likely influences outcomes during influenza active periods (IAP), we performed additional analyses restricted to the IAP. IAPs were defined as per PHE (now UKHSA) reports using information about the consultation rates for ILI and isolation of the virus from sentinel surveys.^21^

*IIV safety* Vaccinated participants with ≥1 IBD flare in the study period were included. The 2018–2019 influenza cycle was divided into baseline, pre-vaccination and post- vaccination periods. The baseline extended from 1 September 2018 to 15 days pre-vaccination, and from 90 days post-vaccination to the earliest of 31 August 2019, date of leaving GP surgery, date of death or the latest date of data collection. The exposed period extended from vaccination to 90 days later and was further categorised as 0–14 days, 15–30 days, 31–60 days, and 61–90 days post-vaccination. The first categorisation of the exposed period was at 14 days post-vaccination as it takes approximately 2 weeks for the serological response, and this period of immune reconstitution would be most strongly associated with disease activity if such an association exists. The 14-day period immediately preceding vaccination was excluded from the baseline period to minimise confounding due to healthy vaccinee effect, which results in people seeking vaccination only when they are well. A Poisson model conditioned on the number of events adjusted for the seasons as categories defined in line with the Meteorological Office description was fitted to calculate the adjsted incidence rate ratios (aIRR) and 95% CI for each exposure period compared with the baseline period. Data management and analysis were performed in Stata v17, Stata Corp LLC, Texas, USA.

### Patient and public involvement (PPI)

PPI members were involved in selecting the research question. They advised us to use readily available datasets instead of undertaking an expensive primary study.

## Results

**Figure 1:**
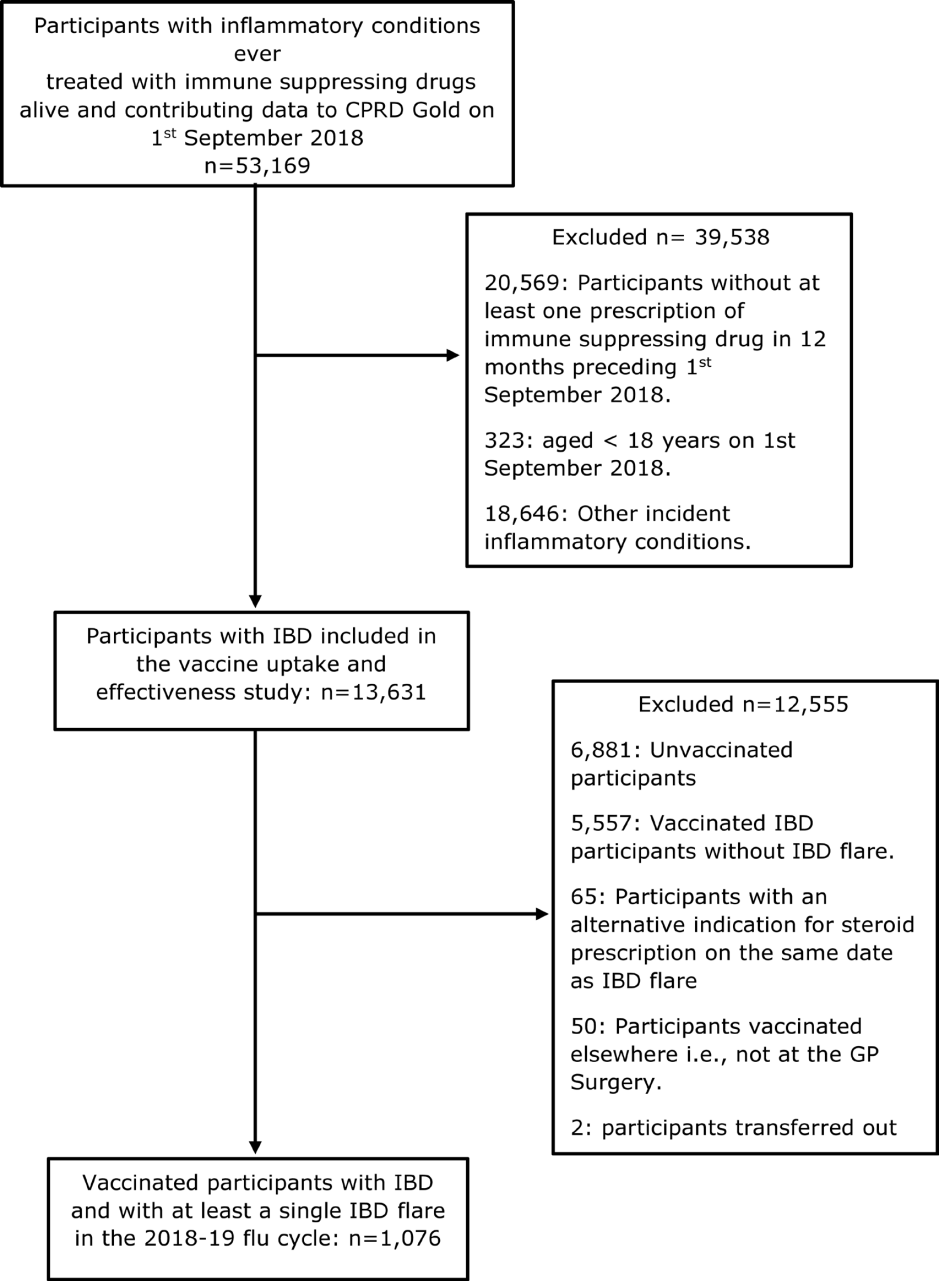
Study population selection criteria.

Data were available for 13 631 people with IBD (figure 1). Of these, 50.4% were male, the mean (SD) age was 52.9 (17.4) years, 45.2% were current or previous smokers, 34.2% had at least one additional at-risk condition for vaccination and 50.1% and 38.4% were previously vaccinated against influenza or pneumococcal vaccinations respectively (table 1).

**Table 1:** Cohort characteristics (n=13 631)

Variable	Distribution
Continuous variables	Mean (SD)
Age	52.9 (17.4)
Body mass index (BMI)	27.1 (5.8)
Missing BMI values	1553 (11.4) *
Charlson’s comorbidity index	0.9 (1.4)
Index of multiple deprivation	3.1 (1.41)
Number of prescriptions^±^	45.8 (57)
Number of consultations^±^	12.1 (10.1)
Number of hospitalisations^±^	0.1 (0.5)
Categorical variables	n (%)
Male sex	6869 (50.4)
*Smoking status*	
Non-smoker	7225 (53)
Current smoker	1546 (11.3)
Ex-smoker	4623 (33.9)
Missing	237 (1.7)
*Alcohol consumption (units/week):*	
Non-drinker	1972 (14.5)
Low drinker (1–14)	7173 (52.6)
Moderate (15–21)	723 (5.3)
Hazardous (>21)	757 (5.6)
Former drinker	803 (5.9)
Missing	2203 (16.2)
Nursing Home resident	86 (0.6)
Previous influenza vaccination	6835 (50.1)
Previous pneumococcal vaccination	5228 (38.4)
Diabetes	1232 (9.0)
Immunosuppression	200 (1.5)
Chronic kidney disease	927 (6.8)
Chronic respiratory disease	2746 (20.2)
Chronic heart disease	586 (4.3)

*Number(percent).

*IIV uptake:* Vaccine uptake during the entire 2018–2019 influenza cycle and before the influenza virus circulated in the community was 49.52% and 32.11%, respectively. Vaccine uptake (95% CI) was 32.56% (31.50–33.64%) in the low-risk groups defined as the under 65s and patients without any additional at-risk condition, and 69.45% (95% CI 68.30 to 70.58%) in the high-risk group defined as patients aged ≥65 years or those with additional at-risk condition. Increasing age, female sex, the presence of an at-risk condition and immunosuppression were independently associated with IIV uptake (table 2).

**Table 2:** Percentage and risk factors of inactivated influenza vaccine uptake in patients with inflammatory bowel disease during the 2018–2019 influenza cycle

	Vaccinated in the entire influenza cycle		Vaccinated in-time before influenza circulation		Incidence rate ratios (IRR)*	
	n	Percent (95% CI)	n	Percent (95% CI)	Crude IRR (95% CI)	Adjusted IRR (95% CI)
Overall	6750	49.52 (48.68,50.36)	4377	32.11 (31.33,32.90)	–	–
Age, years						
< 45	1613	33.47 (32.15,34.82)	994	20.63 (19.51,21.79)	1	1
45–64	2224	43.22 (41.87,44.58)	1461	28.39 (27.18,29.64)	1.29 (1.21,1.38)	1.34 (1.25,1.43)
≥65	2913	79.46 (78.12,80.74)	1922	52.43 (50.81,54.04)	2.37 (2.23,2.52)	2.38 (2.23,2.54)
Sex						
Male	3263	47.50 (46.32,48.69)	2097	30.53 (29.45,31.63)	1	1
Female	3487	51.57 (50.38,52.76)	2280	33.72 (32.60,34.85)	1.09 (1.03,1.14)	1.10 (1.05,1.15)
Additional at-risk conditions†						
Absent	3599	40.14 (39.13,41.15)	2319	25.86 (24.97,26.78)	1	1
Present	3151	67.56 (66.20,68.89)	2058	44.13 (42.71,45.55)	1.68 (1.60,1.77)	1.38 (1.31,1.45)
Immune-suppressing drugs‡						
5-aminosalicylates	4413	45.30 (44.32,46.29)	2877	29.53 (28.64,30.45)	1	1
Immunosuppressants§	2337	60.08 (58.53,61.61)	1500	38.56 (37.04,40.10)	1.33 (1.26,1.39)	1.57 (1.50,1.66)

*Incidence rate ratios for vaccine uptake in the entire influenza cycle;

†There were no patients in with asplenia.

‡latest prescription with 12 months prior to start of 2018-19 flu cycle (01/09/2018)

§Methotrexate, leflunomide, thiopurines, ciclosporin, mycophenolate, tacrolimus, and sirolimus.

*IIV effectiveness:* Primary care consultation for LRTI requiring antibiotics, ILI, hospitalisation due to pneumonia and all-cause death occurred in 294, 38, 45 and 465 people at an incidence rate (95%confidence interval) of 23.35 (20.83,26.18), 2.98 (2.17,4.10), 17.47 (13.05,23.40) and 36.46 (33.29,39.92) per 1000 person-years, respectively. PS was calculated after imputation of 1553 (11.4%), 237 (1.7%) and 2203 (16.2%) missing values on smoking, alcohol consumption and BMI, respectively. Covariate balance between the influenza unvaccinated and vaccinated IBD patients was achieved following IPTW on PS (table 3).

**Table 3:** Covariate balance before and after inverse probability of treatment weighting using the propensity score*

	Vaccinated (n=6750)	Unvaccinated (n=6881)	*d*† before IPTW	*d*† after IPTW
Continuous covariates; mean (SD)				
Age	59 (18)	47 (15)	0.722	−0.067
Body mass index	27.4 (6)	26.5 (5.7)	0.166	−0.025
Charlson’s comorbidity index	1.22 (1.61)	0.49 (1)	0.552	−0.055
Index of multiple deprivation	3.1 (1.4)	3.1 (1.4)	0.027	0.004
Number of prescriptions‡	61.5 (66.8)	30.4 (39.7)	0.565	−0.087
Number of consultations‡	14.8 (11)	9.4 (8.3)	0.546	−0.035
Number of hospitalisations‡	0.1 (0.5)	0.1 (0.4)	0.076	−0.050
Categorical covariates; n (%)				
Male	3263 (48.3)	3606 (52.4)	0.081	0.002
*Smoking status*				
Current smoker	639 (9.5)	937 (13.6)	0.130	0.008
Ex-smoker	2652 (39.3)	2031 (29.5)	0.207	−0.004
*Alcohol consumption (units/week):*				
Low drinker (1–14)	4190 (62.1)	4382 (63.7)	0.033	0.009
Moderate (15–21)	408 (6)	462 (6.7)	0.027	0.005
Hazardous (>21)	380 (5.6)	516 (7.5)	0.076	−0.003
Former drinker	554 (8.2)	375 (5.5)	0.109	−0.012
Nursing Home	59 (0.9)	27 (0.4)	0.061	−0.024
Previous influenza vaccination	5648 (83.7)	1187 (17.3)	1.777	−0.011
Previous pneumococcal vaccination	3988 (59.1)	1240 (18)	0.930	−0.016
Diabetes	960 (14.2)	272 (4)	0.363	−0.030
Immunosuppression	150 (2.2)	50 (0.7)	0.124	−0.032
Chronic kidney disease	735 (10.9)	192 (2.8)	0.325	−0.058
Chronic respiratory disease	1677 (24.8)	1069 (15.5)	0.233	0.002
Chronic heart disease	489 (7.2)	97 (1.4)	0.290	−0.001

*Data from one imputed dataset.

†Standardised difference; .

‡In previous 12 months.

The IIV protected from all-cause death (aHR (95% CI): 0.73 (0.55,0.97). There was no association between vaccination with the IIV and hospitalisation for pneumonia (aHR (95%confidence interval) 0.52 (0.20,1.37) primary care consultation for LRTI (aHR(95%confidence interval): 1.42 (0.98 to 2.06)) and ILI (aHR (95%confidence interval): 1.42 (0.59 to 3.41)). These findings remained unchanged when the follow-up time was restricted to the IAP (table 4).

**Table 4:** Inactivated influenza vaccine effectiveness in people with inflammatory bowel disease

Outcomes	Vaccinated	Entire influenza season					Influenza active period
		Number of events	Person-years	Event rate (95% CI)/ 1000 person-years	Unadjusted HR (95% CI)	Adjusted HR* (95% CI)	Adjusted HR* (95% CI)
Primary care consultation for LRTI requiring antibiotics	No	145	7233	20.05 (17.04 to 23.59)	1.00	1.00	1.00
	Yes	149	5357	27.82 (23.69 to 32.66)	1.51 (1.18 to 1.94)	1.42 (0.98 to 2.06)	1.46 (0.98 to 2.16)
Primary care consultation for ILI	No	19	7289	2.61 (1.66 to 4.09)	1.00	1.00	1.00
	Yes	19	5446	3.49 (2.23 to 5.47)	1.18 (0.61 to 2.27)	1.42 (0.59 to 3.41)	1.47 (0.60 to 3.64)
Hospitalisation for pneumonia	No	19	1525	12.46 (7.95 to 19.53)	1.00	1.00	1.00
	Yes	26	1051	24.75 (16.85 to 36.35)	1.96 (1.04 to 3.70)	0.52 (0.20 to 1.37)	0.48 (0.18 to 1.27)
All-cause death	No	155	7298	21.24 (18.15 to 24.86)	1.00	1.00	1.00
	Yes	310	5457	56.80 (50.82 to 63.49)	2.29 (1.88 to 2.78)	0.73 (0.55 to 0.97)	0.77 (0.57 to 1.04)

*IPTW using the propensity score.

*IIV safety:* Data for 1076 vaccinated people with IBD and at least one IBD flare in the study period were included in the analysis (figure 1). The majority were female (53.5%), and their mean (SD) age was 55^17^ years. 581 (54%) had UC, 339 (31.5%) had Crohn’s disease and 156 (14.5%) had IBD without any specific coding for subtype. 906 (84.2%), 162 (15.1%) and 8 (0.7%) participants had one, two and three IBD flares, respectively. 19 participants (1.8%) did not contribute data for the entire follow-up period due to death (n=7 (0.7%)) or transfer out of GP practice (n=12 (1.1%)). Vaccination against seasonal influenza was not associated with IBD flares 90 days post-vaccination (table 5).

**Table 5:** The association between influenza vaccination and inflammatory bowel disease flare

Risk period (days)	Events (n)	IRR (95%confidence interval)	Adjusted IRR (95%confidence interval) *	P value
Baseline	884	1.00	1.00	
15 days pre-vaccination	61	1.17 (0.90 to 1.51)	1.13 (0.84 to 1.52)	0.431
Post-vaccination intervals				
0–90 days	309	0.99 (0.87 to 1.12)	0.68 (0.46 to 1.02)	0.060
0–14 days	51	0.97 (0.73 to 1.29)	0.92 (0.72 to 1.36)	0.941
15–30 days	55	1.05 (0.80 to 1.38)	0.99 (0.72 to 1.36)	0.941
31–60 days	99	0.95 (0.77 to 1.17)	0.87 (0.62 to 1.21)	0.409
61–90 days	104	1.00 (0.81 to 1.22)	0.90 (0.63 to 1.29)	0.576

*adjusted for Season.

## Discussion

This large nationwide-wide study found that the IIV uptake is low in people with IBD, even among those with an additional indication for vaccination, and a substantial proportion of those vaccinated did not receive optimally timed vaccination before the influenza virus circulated in the community. However, the vaccine did not produce excess IBD flares. IIV was protective against all-cause death although this could be due to unmeasured confounding.

Our estimate of 50% IIV uptake is comparable to those observed in previous questionnaire surveys. In the USA, North America and Europe, influenza vaccine uptake ranged between 28.1% and 61.4%.^5–9^ Our data predate the COVID-19 pandemic. There are no studies of IIV uptake during the post-pandemic era in people with IBD. In the UK general population, the uptake of influenza vaccine has increased in the post-pandemic era.^22^ Similarly, the uptake of influenza vaccine in 2018 was expectedly lower than that of vaccination against COVID-19 in people with IBD which ranged between 71% and 80%.^23^ Unlike previous studies of influenza vaccination uptake limited by the small sample size and use of survey questionnaire prone to recall bias, our findings are from the CPRD, a large primary care database, suited for this study, as vaccination in the UK is administered in primary care. As reported in people with autoimmune rheumatic diseases, the vaccine uptake was higher in women than men and increased with age.^17 24^ Unsurprisingly, the presence of comorbidities and immunosuppressive drug treatment was significantly associated with vaccine uptake as reported in people with autoimmune rheumatic diseases.^24^

Immunosuppressant (including biologic) use was associated with an increased vaccine uptake in most studies included in a systematic review.^25^ However, the use of biologics and other immunosuppressive drugs was negatively associated with vaccination with live herpes zoster vaccine in one study as would be expected for a live virus vaccine and unexpectedly, negatively associated with vaccination with seasonal influenza vaccine in another study included in this review.^26 27^

Vaccination with the IIV was not associated with an increased risk of IBD flare in this study. This is consistent with uncontrolled data from previous studies.^28–30^ In an observational study of 575 IBD patients on immunomodulators or anti-tumor necrosis factor (TNF)-alpha, vaccinated between November 2009 and March 2010 in 14 European countries, the H1N1 vaccine was found to be well tolerated in terms of disease control without flares in over 96% patients.^30^ In a randomised controlled trial of 137 subjects with IBD on maintenance infliximab therapy allocated to receive the 2012/2013 IIV at different time points with respect to the infliximab infusion, adverse effects lasting longer than 24 hours were infrequent, and there were no severe adverse effects requiring medical attention.^29^ Similarly, COVID-19 vaccine was not associated with IBD flares in a previous study.^31^

In this study, we used data from the 2018–2019 influenza vaccination cycle. Nevertheless, we believe that the findings of no association between vaccination and IBD flare are applicable to future seasonal influenza vaccination cycles because the influenza antigen in a vaccine only varies slightly from year to year to account for the antigenic drift. This is unlikely to be substantial enough to cause differences in the association between vaccination against seasonal influenza and IBD flares in different years. Even though the antigens are varied each year in seasonal influenza vaccines, the available seasonal influenza vaccines are all protein subunit modality, and the antigen content of vaccines is standardised for regulatory reasons @ 15 mcg of haemagglutinin per influenza strain (of which, there are four in a standard quadrivalent vaccine). Most of the immunological side effects of vaccination are attributed to adjuvants that do not vary from year to year.

In this study, IIV was not associated with a statistically significant reduction in respiratory morbidity. Reasons for this could include the use of surrogate outcomes such as pneumonia and LRTI treated with antibiotics rather than laboratory-confirmed influenza, and poor protection against infection in adults for influenza A(H3N2) in the 2018–2019 influenza cycle.^32^ Thus, further research on this topic is warranted. Immunologic studies in children and adults with IBD found IIV to generally induce appropriate immune response to influenza.^33 34^ However, when patients are receiving immunosuppressive therapies with combined thiopurines and anti-TNF-alpha agents, serologic response to vaccines is lower compared with monotherapy or non-immune suppressing treatment.^28 34 35^ Nonetheless, even a blunted vaccination response is thought to be benefi^36^cial, and immunological correlates of protection against influenza remain poorly understood.^32^

Strengths of this study include the generalisability of its findings to patients with IBD in the UK due to its data source, CPRD, a representative of over 98% of the UK population registered with a GP surgery.^15^ We used a combination of diagnostic and prescription codes to identify people with IBD, increasing the validity of our case definition. Studies of VE are biased due to confounding by indication and healthy user bias, but we attempted to account for this using PS for vaccination and employing inverse probability treatment weighting on the PS in the Cox regression analysis. Nevertheless, it is possible that our results are influenced by unmeasured confounding. The SCCS methodology that is widely used in vaccine safety studies^37^ ensures that non time-dependent between-person confounding was excluded because participants were compared only with themselves at different time points.^38^ There was no selection bias because all patients who had received both vaccination and experienced an IBD flare were included in the IIV safety analysis.

However, this study has several limitations. First, we could not include laboratory-confirmed influenza as an outcome as routine viral testing is not conducted for patients presenting to GPs. For this reason, we included ILI as an outcome—this could include many other viral illnesses that are not preventable by vaccination against influenza. Second, we were unable to assess the association between vaccination and death due to pneumonia because of only 11 events. Third, vaccinations occurring outside the GP surgery are not recorded in the CPRD. This biases the VE results towards null rather than inflating estimates and could explain the lack of significant protective effects on respiratory morbidity and mortality. Fourth, we did not have access to information on hospitalisations across the UK because HES data are available for only NHS hospitals in England.^39^ Fifth, we were unable to assess the impact of biologics on IIV safety because their prescription is not recorded in the CPRD as they are hospital prescribed. According to the UK IBD audit,^40^ just over 20% people with IBD were prescribed a biologic (±conventional steroid sparing drug) in the UK. While those prescribed a biologic in combination with conventional steroid-sparing drug were included in the study by virtue of the latter drug being prescribed from primary care, those prescribed biologic monotherapy were excluded from the study population. The proportion of people with IBD prescribed biologic monotherapy in the UK is unknown. As vaccination is promoted proactively in people treated with biologics, it is likely that their exclusion reduced the estimated vaccine uptake. Their exclusion may also have reduced the number of disease flares ascertained in the study population, thereby reducing the absolute incidence rate of flares. However, there is no reason to suspect that this will affect the temporal relationship between vaccination and disease flares as this is assessed using a rate ratio. We see no reason though to expect more extreme immunologically driven side effects in these groups given that IIV is less immunogenic with biologic use.^29^ Nevertheless, further studies are required to evaluate whether vaccination against seasonal influenza is temporarily associated with IBD flares in people treated with biologics. Sixth, because our definition of IBD flare was based on corticosteroid or 5-ASA prescription, minor flares not needing drug treatment were excluded. It is possible that there may be an association with minor flares that were not confirmed; on balance, such effects would be unlikely to greatly discourage vaccination uptake and it is the more significant flares that we have studied which are of primary concern. Seventh, because we considered a single influenza season, our study is limited by low power. Eighth, our data pre-date the COVID-19 pandemic, and because of the increased awareness about vaccination since the pandemic, uptake of influenza vaccine may have improved and should be evaluated in people with IBD. Finally, because 5-ASAs are not immunosuppressive, it is possible that some people included were not eligible for vaccination. However, even in those prescribed an immune-suppressive treatment, the vaccine uptake was low at 60%.

In conclusion, this study provided the first UK-wide population-based evidence that the uptake of vaccination against influenza is low in people with IBD and that vaccination does not occur in time before the virus circulates in the community in this population. Vaccination against influenza was not associated with IBD flare. These data should be used to promote vaccination in IBD population.

## Data Availability

Data may be obtained from a third party and are not publicly available. Data used in the study are from the Clinical Practice Research Datalink (CPRD). Due to CPRD licencing rules, we are unable to share data used in this study with third parties. The data used in this study may be obtained directly from the CPRD. Study protocol is available from www.cprd.com.
